# Forced-Air Warming Provides Better Control of Body Temperature in Porcine Surgical Patients

**DOI:** 10.3390/vetsci3030022

**Published:** 2016-09-09

**Authors:** Brian T. Dent, Karla A. Stevens, Jeffrey W. Clymer

**Affiliations:** 1 College of Veterinary Medicine, The Ohio State University, Columbus, OH 43210, USA; dent.63@osu.edu; 2 Ethicon, Inc., Cincinnati, OH 45242, USA; ksteven5@its.jnj.com

**Keywords:** porcine, surgery, body temperature, hypothermia, resistive blanket, forced air heating

## Abstract

*Background:* Maintaining normothermia during porcine surgery is critical in ensuring subject welfare and recovery, reducing the risk of immune system compromise and surgical-site infection that can result from hypothermia. In humans, various methods of patient heating have been demonstrated to be useful, but less evaluation has been performed in techniques to prevent hypothermia perioperatively in pigs. *Methods:* We compared body temperature regulation during surgery before and after modification of the ambient temperature of the operating laboratories. Three different methods of heating were then compared; a standard circulating water mattress, a resistive fabric blanket, and a forced hot air system. The primary measure was percentage of temperature readings outside a specification range of 36.7–40.0 °C. *Results:* Tighter control of the ambient temperature while using a circulating water mattress reduced the occurrence of out-of-specification body temperature readings from 20.8% to 5.0%, with most of these the result of hypothermia. Use of a resistive fabric blanket further reduced out-of-specification readings to 1.5%, with a slight increase in the occurrence of hyperthermia. Use of a forced air system reduced out-of-specification readings to less 0.1%. *Conclusions:* Maintenance of normothermia perioperatively in pig can be improved by tightly controlling ambient temperatures. Use of a resistive blanket or a forced air system can lead to better control than a circulating water mattress, with the forced air system providing a faster response to temperature variations and less chance of hyperthermia.

## 1. Introduction

Perioperative hypothermia is a recurrent challenge which is critically important to patient welfare, recovery, and thus, reliable data collection. As such, it must be proactively and thoroughly addressed. Hypothermia occurs in nearly all surgical patients who do not receive supplemental heat, primarily as a result of the cooler operating room temperatures required for surgeon and staff comfort along with the thermoregulatory impairment experienced by patients under anesthesia [1,2,3,4]. Anesthetic-induced tonic vasoconstriction inhibition leads to peripheral vasodilation, allowing the rapid redistribution and loss of core body heat within the first hour of surgery [2]. Approximately three hours into surgery, the linear decline in core temperature reaches a plateau in response to the development of a thermal equilibrium or a sufficiently hypothermic state which reactivates peripheral vasoconstriction [5,6,7]. Under such conditions, tissue oxygenation is significantly reduced along with leukocyte migration, neutrophil phagocytosis, as well as antibody and cytokine production [8,9]. Given such immunocompromised conditions at the tissue level, it is unsurprising that hypothermic patients experience higher wound infection rates [10,11,12]. One retrospective review of over 500 laparotomies identified a 221 percent increase in surgical site infections in its hypothermic patients [13]. In addition, hypothermia-induced catecholamine release and systemic vasoconstriction can dramatically increase blood pressure [14], cardiac demand, and subsequently lead to increased incidence of myocardial ischemia [10]. Uncorrected perioperative hypothermia has also been demonstrated to prolong the duration of pharmaceutical action [15] and induce coagulopathy [16], as in a recent review of intraoperative warming trials which identified a more than 60 percent increase in the mean surgical blood loss in patients not receiving supplemental heat [17].

At Ethicon, the concern of hypothermia is paramount in porcine surgical patients which are relied upon to produce dependable, consistent results regarding the safety and efficacy of experimental medical devices. Though perioperative hypothermia has not been extensively studied in pigs, it has been demonstrated to reduce systolic and diastolic cardiac function as well as myocardial compliance in porcine and canine models [18,19,20]. In order to maintain normothermia in pigs, between 38 and 39.5 °C [21], the heat loss associated with surgery must be counterbalanced by externally supplied heat. Such supplemental heat can come from a variety of patient warming systems; including forced-air, resistive fabrics, and circulating hot water mattresses. The most widely utilized perioperative warming systems provide forced-air heating, the effectiveness and safety of which have been well reported [22,23]. However, disposable forced-air covers can generate prohibitive long-term cost, indicating a potential benefit of non-disposable warming technologies. Conductive blankets and circulating water mattresses are reusable heat sources, but various human studies have demonstrated a lack of consensus regarding the efficacies of available products [17]. Product evaluation for the purpose of maintaining porcine perioperative normothermia has not, to the authors’ knowledge, been previously described.

This study investigated the incidence of hypothermia in anesthetized porcine surgical patients under a standard warming protocol and evaluated the efficacy of various warming methods, including ambient temperature adjustment as well as the use of circulating hot water mattresses, conductive heating blankets, and forced-air warming systems.

## 2. Materials and Methods

**Animals.** Domestic pigs (*Sus scofa domesticus*), cross-breeds of Yorkshire and Hampshire, weighing 35–55 kg were utilized in the development and evaluation of surgical devices. Though the pigs included in this experiment were purchased from different suppliers, all animals shared a vaccination history of *Erysipelothrix rhusiopathiae*, several serovars of *Leptospira*, and *Parvovirus.* All animals are brought onsite and housed according to the Association for Assessment and Accreditation of Laboratory Animal Care International guidelines for a minimum of a seven day quarantine period with other pigs from the same vendor prior to their use. Pigs were housed in pairs in slotted-floor cages and maintained on a 12:12-h light: dark cycle at 22 ± 5 °C and 30%–70% relative humidity, with room air changes occurring at least ten times every hour. During this time, they were fed two scoops of Purina Mills Lab Porcine Grower Diet once daily. Water was provided *ad libitum* and enrichment devices were made available. All pigs were examined by the attending veterinarian prior to their procedures and any pig determined to be unfit for surgery was removed from study.

**Surgical procedures.** All procedures were covered by protocols reviewed and approved by the Ethicon Institutional Animal Care and Use Committee. In the spirit of our 3R’s program (reduction, refinement, replacement) to improve animal testing, we sought to make best use of data already acquired, hence our control was historical data. All included surgeries were part of an educational course which repeats periodically, thus maintaining a relatively standardized set of surgery parameters throughout the year. Surgeries covered in this program consist of various abdominal and thoracic surgeries approximately split between open and laparoscopic access. Duration of surgeries were at least 3 hours and up to 6 hours and all animals were euthanized post-operatively. Anesthesia was induced with an intramuscularly administered mixture of 5 mg/kg telazol and xylazine, (Telazol, Rompun, Lloyd Inc., Shenandoah, IA, USA) and 0.01 mg/kg glycopyrrolate (Robinul, Baxter Labs, Deerfield, IL, USA); anesthesia was maintained with 1.5%–3.0% isoflurane gas with a target minimum alveolar concentration of 1.5%.

**Ambient temperature.** Four different surgical suites were utilized. Historical ambient temperature measurements were used from each suite six times a day for nine days for a baseline evaluation. Temperature ranges and averages were determined for each suite and compared to a standard operating room temperature of 20–22.8 °C, as recommended by the Association of periOperative Registered Nurses and the American Institute of Architects [24]. As needed, surgical suite temperatures were adjusted to within the acceptable range and verified for one month prior to the collection of patient temperature data.

**Body temperatures prior to ambient adjustment.** A historical review of porcine surgeries within the Ethicon facility in 2010 (n = 159) was utilized to determine baseline hypothermia rate. For the purposes of this study, porcine hypothermia was defined as a core body temperature below 36.7 °C (98.0 °F). Historically, we have observed serious physiological changes at body temperatures below 96 °F (35.6 °C), so we set our action level for hypothermia 2.0 °F (1.1 °C) higher. According to the standard operating procedures, any animals which would have experienced hypothermia during these surgical laboratories would have been supplemented with heat from a circulating water mattress placed below the patient pre-operatively. However the recovery from hypothermia was typically slow or incomplete, leading to our search for improved methods of body temperature control. Intraoperative temperature data, taken with a calibrated nasal probe (Cardiocap 5 Monitor with Central Temperature Probe 165612, Datex-Ohmeda, Madison, WI, USA) inserted until contact is made with the nasal turbinate at the time of anesthetic induction, was collected from the records of 160 pigs at a consistent time point three hours into surgery.

**Body temperatures post ambient adjustment.** The effect of the tighter control of ambient temperature on body temperature was determined through the intraoperative sampling of 32 pigs three hours into surgery. Timing of sampling was based on prior experience that hypothermia was infrequently observed during the first three hours of surgery. All incidents of hypothermia (i.e., <36.7 °C) were addressed using a circulating hot water mattress (Temp Pump Professional TP700 with Temperature Therapy Pad TP22G, Gaymar Industries, Inc., Orchard Park, NY, USA) placed under the animal per the established warming protocol in effect for the surgical labs included in the in-house historical review.

**Body temperatures with supplemental heating.** Once a new frequency of hypothermia was determined after the ambient temperature changes, intraoperative temperature measurements were taken for surgeries being supplied supplemental heat from a conductive fabric warming blanket (n = 19, HotDog Patient Warming System, Augustine Temperature Management, Eden Prairie, MN, USA) or a forced-air warming system (n = 25, Bair Hugger Animal Health Warming Unit 50577, 3M, Maplewood, MN, USA). The Bair Hugger was used without a perforated mattress by inserting the output tube under a standard surgical drape that cocooned the animal. When in use, the Bair Hugger was set to Medium (100 °F /38 °C) or High (110 °F /43 °C). Both devices were used in a similar distribution of open and laparoscopic abdominal and thoracic surgeries.

### Statistical Analyses

Primary measures were parametric estimates of the percentage of temperature readings outside of control limits. For ambient temperature, the control range was 20.0–22.8 °C. For porcine body temperature, the control range was 36.7–40.0 °C. For both the ambient temperature and body temperature measurements, we determined the percent occurrence of out-of-specification temperatures using the mean and standard deviation.

Based on the near normal distribution of data, comparisons of means before and after adjustments were performed using Student’s t-test, and comparison of standard deviation using the F-test, with α = 0.05. For ambient temperature measurements, we compared the historical baseline mean and standard deviation to adjusted ambient temperature measurements during use of the circulating hot water mattress, the HotDog Patient Warming System and the Bair Hugger Animal Health Warming Unit. For body temperature measurements, we compared means of the historical baseline temperatures to after ambient temperature adjustment both with the use of the hot water mattress, and then compared both HotDog and Bair Hugger to the hot water mattress values.

## 3. Results

**Figure 1:**
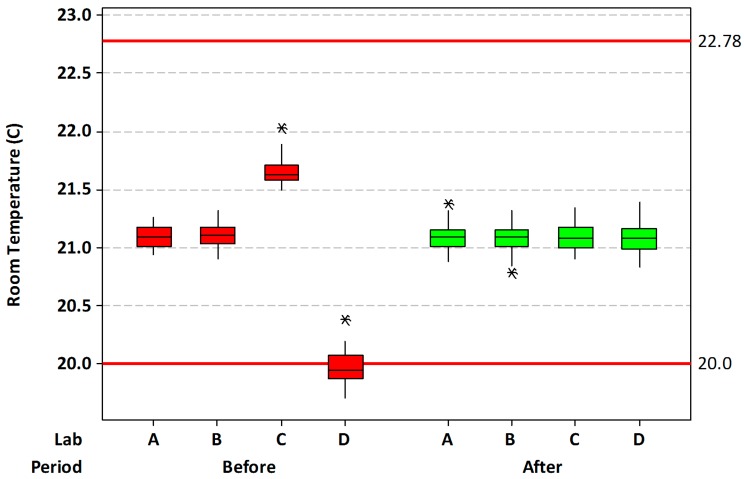
Ambient room temperature of the four rooms before and after temperature control adjustments. The control limits are those recommended by the Association of periOperative Registered Nurses (AORN). Asterisks (*) in the graph represent outlier points, i.e., at least 1.5 times the interquartile range; these points were not excluded from the analysis.

**Ambient temperature**. Baseline ambient temperature across the four surgical suites utilized in this study had an average value of 21.0 ± 0.62 °C, with a range of 19.7–22.0 °C. Most of the lowest data was collected from one particular suite (Figure 1). Following adjustment of the ambient temperatures, the average was 21.1 ± 0.11 °C, with a range of 20.8–21.4 °C. The average was slightly, but significantly increased (*p* = 0.005), while the overall standard deviation was greatly reduced (*p* < 0.001). Using the recommended AORN temperatures (20.0–22.78 °C) as limits, the percentage of out-of-specification readings decreased from a baseline value of 6.4% to 0.0% after room temperature adjustments.

**Body temperatures prior to ambient adjustment.** Initial in-house historical review of temperature data from 159 porcine surgeries supplemented with heat from a circulating hot water mattress revealed an estimated 20.4% hypothermia incidence with a mean intraoperative temperature of 37.4 °C and a low incidence (0.4%) of hyperthermia (Table 1). The total percentage of out-of-specification body temperatures (hypothermia and hyperthermia) was 20.8%.

**Body temperatures post ambient adjustment.** Among pigs that underwent surgery in the newly temperature-regulated suites, still receiving traditional circulating water heat as needed, there was an increase in mean body temperature of +0.6 °C (*p* = 0.002) with hypothermia incidence decreased to 4.6%, a five-fold reduction (Table 1). Hyperthermia occurrence remained at 0.4%, for a total out-of-specification rate of 5.0%. This improvement from merely adjusting the room temperature met our initial target for hypothermia, however, direct control of the individual animal’s environment offered the prospect of complete compliance with the control limits. Hence the conductive fabric (HotDog) and forced-air (Bair Hugger) warming systems were compared to the standard circulating water mattress.

**Figure 2:**
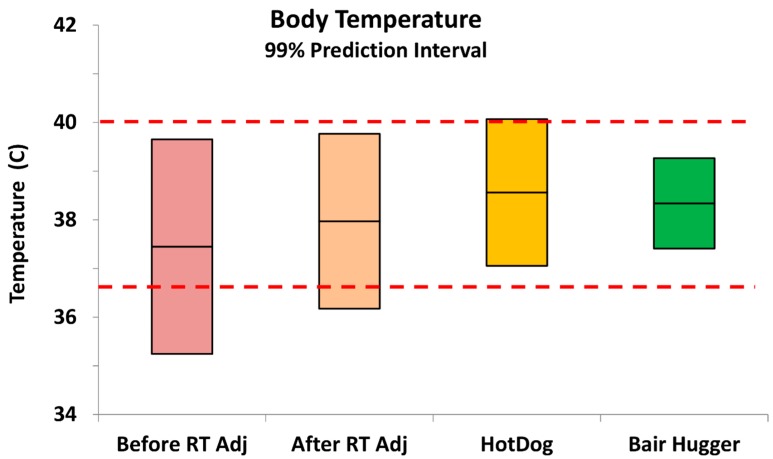
Intraoperative porcine body temperatures during surgery before and after room temperature adjustments, and after room temperature adjustments with addition of a supplemental heat source. For the first two measurements a circulating hot water mattress was used. Without use of a supplemental heat source there were occurrences of hypothermia. With the HotDog warmer there were a few cases of hyperthermia. There was no hypothermia or hyperthermia for the Bair Hugger. The middle line of each box is the mean value, and the range represents the 99% prediction interval from a parametric estimation.

**Body temperatures with supplemental heating.** For pigs that received intraoperative heat supplementation (Table 1, Figure 2) from the HotDog conductive fabric system, mean body temperature increased by +0.6 °C compared to the standard circulating water mattress (*p* = 0.005) with 0.16% hypothermia and 1.3% hyperthermia, with hyperthermia being considered body temperatures above 40.0 °C. Hence the total out-of-specification results were less than 1.5%. For pigs supplemented with the Bair Hugger forced-air system the mean body temperature increased +0.3 °C compared to the circulating water mattress (*p* = 0.025) with 0.0% incidence of hypothermia and hyperthermia.

## 4. Discussion

Though there has been much inconsistency in the results of numerous studies comparing various warming systems, these studies have not previously been conducted in porcine surgical models, hence for our review we used studies primarily performed in humans. While our results seems to correlate with a number of publications supporting the use of forced-air warming systems [25,26], the differences between our results and other apparently contrary preceding work [27,28] are difficult to compare directly, given the dissimilar circumstances of these experiments such as skin conductivity, body positioning of the warming apparatus and total contact surface area, as well as the difference between preventative and corrective use of the devices.

In the development of surgical equipment, the importance of having a reliable porcine surgical model is paramount, ensuring the consistency and validity of data which is generated and used to endorse the development of human surgical instruments and techniques. Anesthesia and operating conditions tremendously predispose subjects to intraoperative hypothermia, which can present a very significant dilemma, both to the process of data collection and the maintenance of animal welfare throughout experimentation. Of paramount importance in complying with animal welfare protocols is the commitment to reducing and eliminating any unnecessary animal use in the laboratory. As a result, many of the pigs which undergo surgery at our facility are under anesthesia for prolonged periods of time, over five hours on average, in order to maximize the usefulness of every specimen. This fact only increases the likelihood of intraoperative hypothermia, which has been demonstrated to occur more frequently in human subjects under anesthesia for longer than three hours [29].

Our initial retrospective review determined that there was, in fact, a substantial incidence of hypothermia among our porcine subjects, as was anticipated based on the widespread incidence in human surgical patients reported in the literature. This lends credence to the application of conclusions regarding human anesthesia complications to our porcine model as well as indicates the ineffectiveness of our traditional patient warming protocol, the circulating water mattress.

Additionally, we found that in our institute a simple and effective means for reducing, though not eliminating, intraoperative hypothermia was by adjusting the ambient temperature of the operating suite to the recommended range. The cost of this temperature change is insignificant, as the additional spending required for heat in the winter is partially compensated by savings on cooling in the summer.

This considerable reduction in hypothermia incidence indicates a cost-effective means of better ensuring the welfare of most surgical patients; however, the inherent predisposition to hypothermia makes it improbable that the condition will be avoided completely with such simple corrective measures. Furthermore, it does not circumvent the need for reliable means of resolving hypothermia in those remaining patients that do experience it.

With regard to the most efficacious warming system, our results point to forced-air warming as the method of choice, as it completely eliminated hypothermia from our surgical patients. Although the conductive fabric system reduced the occurrence of hypothermia, an unexpected result was the development of hyperthermia in some cases, which was unprecedented with the circulating water mattress. While it was likely that the relatively superior warming abilities of the other two warming systems predisposed them somewhat to the possibility of inducing hyperthermia, this effect was not observed with the forced-air system despite producing a higher mean body temperature than the circulating water mattress. One apparent explanation for this is the more immediate and precise temperature control provided by forced-air systems, as opposed to the more gradual temperature change in the resistive blankets. Often, technician overestimation of patients’ supplemental heat requirements leads to the blankets being turned to higher than necessary temperatures from which the heat dissipates too slowly to be able to quickly adjust patient temperature back down. This, in turn, leads to the larger range of temperature fluctuations seen with this device. In contrast, forced-air systems are better equipped to prevent this problem through the option of blowing cool air over the patient to more rapidly dissipate any excess heat.

While one might consider the potential for turbulent air movement while using forced-air warming to cause increased surgical site contamination, it has been demonstrated in multiple studies to reduce post-operative infection rates [8,12], probably due to the physiologic effects of its superior body temperature maintenance. This potential obstacle is somewhat beyond the scope of our investigation however, as all of the surgeries included were acute and neither patient survival nor post-operative complications were considered.

A limitation of this study is that it was performed retrospectively without the benefit of a controlled design. Hence we cannot be certain whether other factors varied between the treatments, and these factors may have influenced the outcomes in an unknown fashion. However, since the time of these initial observations, we have continued to use a forced air system with success, so we suspect that the actual treatments used in the study were the most critical. Additionally, we have not observed body temperature in recovering animals, since all subjects were euthanized immediately after surgery. Again, forced airs systems appear to be useful during recovery in survival studies. Furthermore, there were a variety of procedures performed and it is generally observed that open procedures, depending upon the size of the incision, would require more attention to body temperature than laparoscopic ones. The distribution of procedures was similar during the different phases of the study, so we feel the comparisons between conditions/devices were, in general, fair.

## 5. Conclusions

Overall our findings indicate that careful monitoring of ambient conditions and use of forced-air warming can reduce the incidence of hypothermia during porcine surgery, and this is expected to result in less patient morbidity and faster recovery. Since the completion of this study, our laboratory has continued to use forced-air warming for intraoperative heat supplementation of its porcine patients. Hypothermia incidence was monitored for several months following the study conclusion with no reported morbidity.

## Figures and Tables

**Table 1:** Summary of temperature measurements.

Measure	Before RT Adj. (Baseline)	After RT Adj.	HotDog	Bair Hugger
Room Temperature				
Mean ± St. Dev.	21.0 ± 0.6 °C	21.1 ± 0.1 °C	21.0 ± 0.1 °C	21.2 ± 0.1 °C
Range	19.7–22.0 °C	20.8–21.4 °C	20.7–21.3 °C	20.8–21.6 °C
% Out-of-Spec (20.0–22.8 °C)	6.4%	0.0%	0.0%	0.0%
t-test, Mean vs. Baseline	-	*p* = 0.005	*p* = 0.007	*p* = 0.004
F-test, St. Dev. vs. Baseline	-	*p* < 0.001	*p* < 0.001	*p* < 0.001
Body Temperature n	159	32	19	25
Mean ± St. Dev.	37.4 ± 0.9 °C	38.0 ± 0.8 °C	38.6 ± 0.6 °C	38.3 ± 0.4 °C
% Hypothermic (<36.7 °C)		4.6%	0.2%	0.0%
% Hyperthermic (>40.0 °C)	20.4%	0.4%	1.3%	0.0%
t-test, Mean	0.4%	*p* = 0.002	*p* = 0.005	*p* = 0.025
	-	vs. Baseline	vs. After Adj.	vs. After Adj.
